# Identification of Insulin Receptor Splice Variant B in Neurons by *in situ* Detection in Human Brain Samples

**DOI:** 10.1038/s41598-018-22434-2

**Published:** 2018-03-06

**Authors:** Brian Spencer, Logan Rank, Jeff Metcalf, Paula Desplats

**Affiliations:** 1 Department of Neurosciences, School of Medicine, University of California - San Diego, La Jolla, CA 92093 USA; 2 Department of Pathology, School of Medicine, University of California - San Diego, La Jolla, CA 92093 USA

## Abstract

Insulin and its receptor are widely expressed in a variety of tissues throughout the body including liver, adipose tissue, liver and brain. The insulin receptor is expressed as two functionally distinct isoforms, differentiated by a single 12 amino acid exon. The two receptor isoforms, designated IR/A and IR/B, are expressed in a highly tissue and cell specific manner and relative proportions of the different isoforms vary during development, aging and disease states. The high degree of similarity between the two isoforms has prevented detailed studies as differentiation of the two isoforms by traditional immunological methods cannot be achieved. We describe here a new *in situ* RT-PCR/ FISH assay that allows for the visualization of IR/A and IR/B in tissue along with tissue specific markers. We used this new method to show for the first time that IR/A and IR/B are both expressed in neurons in the adult human brain. Thus, we present a method that enables the investigation of IR/A and IR/B insulin receptor isoform expression *in situ* in various tissues.

## Introduction

Insulin is a major regulator of glucose homeostasis in the body, and while liver, adipose tissue and muscle are its major targets, insulin receptors (IR) have been detected in a wide variety of tissues including: placenta, heart, kidney, hematopoetic cells and the brain (reviewed in ref.^1^). Insulin binding to the insulin receptor (IR) results in auto-phosphorylation and downstream signaling through the insulin receptor substrate (IRS) proteins to MAPK and AKT pathways^2^. The IR is expressed as a single polypeptide encoding both the α-subunit and the ß-subunit. Cleavage of this polypeptide produces the transmembrane ß-subunit, which contains the tyrosine kinase domain and Src binding domain^3^. The α-subunit is the extra-cellular domain that binds insulin^3^. Alternative splicing of the full-length transcript at exon 11 yields two isoforms of the α-subunit: IR/A and IR/B. Signaling through Ras-MEK1-ERK pathway increases the levels of splicing factor SRSF1, leading to inclusion of exon 11 and favoring IR/B isoform expression^4^. The two isoforms of IR are expressed in a highly tissue and cell specific manner and relative proportions of the different isoforms vary during development, aging and disease states^5^.

Signaling through insulin receptor occurs through the phosphoinositol-3 kinase (PI3K), however the specific PI3K utilized by each isoform determines downstream signaling cascades^6^. IR/A intracellular signaling occurs through PI3K class 1a, p70 s6 (p70s6k) and IR/B occurs through PI3K class II-like activity and protein kinase B (PKB/c-Akt)^6^. Intracellular signaling through the IR isoforms follows separate pathways leading to different fates. Insulin binding to IR/A triggers the classical mitogenic signaling cascade whereas, binding to IR/B activates the metabolic phenotype pathway^1,7^.

While insulin receptor is ubiquitously expressed in all tissues in the human body, the ratio of IR/A:IR/B favors IR/B in liver, skeletal muscle, adipose tissue and kidney^5,8^. In contrast, in tissues where the insulin metabolic effects are most needed such as fetal or tumorigenic, IR/A is favored^5,8^. However, because each tissue is composed of numerous cell types, the individual expression ratios of IR/A and IR/B on each cell within the tissue has not been determined. In the brain, whereas glial cells have been shown to express IR/A and IR/B *in vitro*, in neurons, only IR/A has been shown to be expressed *in vitro*^9,10^. Further analysis of IR expression of neuronal IR expression appears to show that IR/A is predominantly expressed in immature/progenitor cell populations with little to no IR/B detected *in vivo*^11^. Neuronal-expressed IR/A appears to be differentially glycosylated *in vivo*, further complicating matters^12^.

The IR/B isoform splice variant is differentiated from the IR/A by only a 36 nucleotide (12aa) exon^5^ preventing specific detection by immunological methods. Recently a quantitative real-time PCR technique was developed to differentially quantify IR/A and IR/B transcripts in cells and whole tissues allowing the quantification of each isoform levels^13^. However, this technique does not allow the study of IR/A and IR/B isoforms in a specific cell-type manner.

We describe here the development of an *in situ* RT-PCR/ FISH assay for the differential detection of IR/A and IR/B in cultured cells and in human tissue that, by coupling with immunohistochemistry, allows the specific localization of IR/A and IR/B expression to specific cell types for the first time. This technique will be useful in the study of specific IR isoform expression in a variety of human tissues such as the brain, liver and pancreas to understand their role in development and disease.

## Results

**Figure 1:**
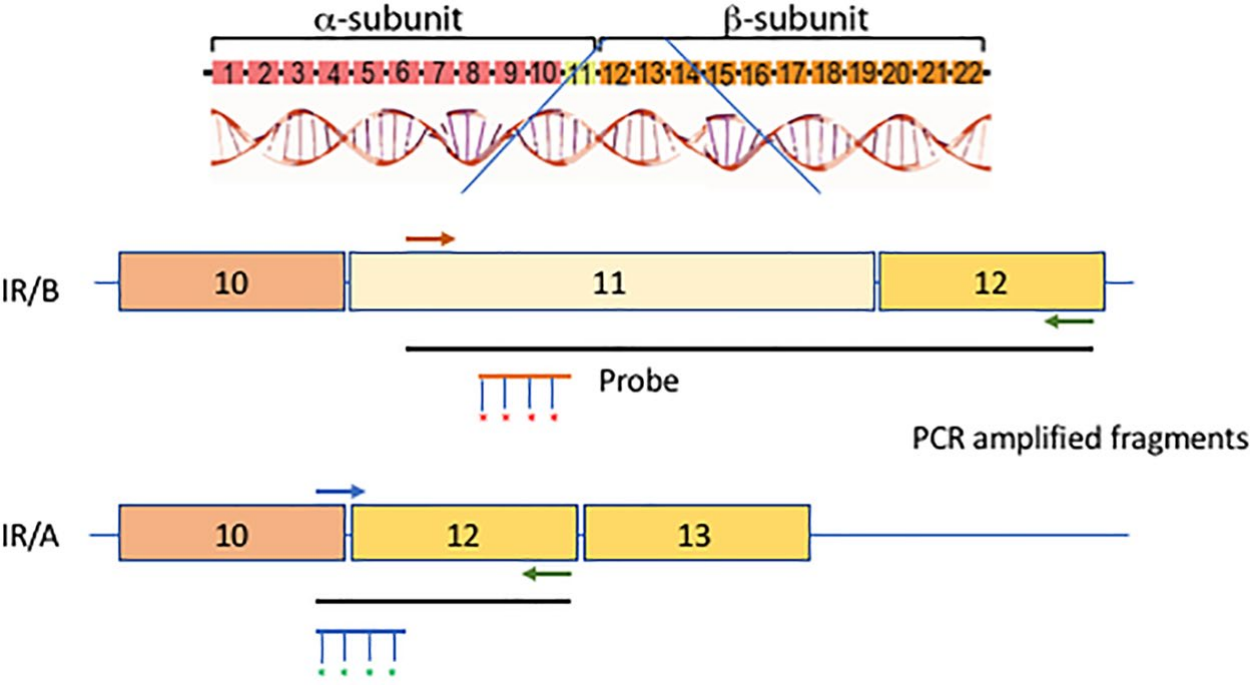
Diagram of *in situ* RT-PCR/ FISH design for insulin receptor isoforms A and B based on exons 10–12 of the coding region of human insulin receptor. Primers^13^ were designed to specifically amplify insulin isoforms A or B (Table 1). Probes labeled with Alexa 594 (IR/B) or Alexa 488 (IR/A) were used in the FISH component of the assay to detect the amplified products.

The IR/B isoform splice variant is differentiated from the IR/A by only a 36 nucleotides (12aa) exon (Fig. 1). We utilized a primer specific for exon 11 that amplified isoform B and a primer spanning the exon 10–12 junction that amplified isoform A^13^ (Fig. 1, Table 1). The use of these primers in Reverse Transcriptase mediated PCR (RT-PCR) along with specific probes designed either for the exon 11 (IR/B) or the exon 10–12 junction (IR/A) allow the specific *in situ* detection of the insulin receptor isoforms (Fig. 1, Table 1).

**Table 1:** Oligonucleotides used for RT-PCR and FISH^13^.

IR/A forward primer	5′ TTT TCG TCC CCA GGC CAT C 3′
IR/B forward primer	5′ CCC CAG AAA AAC CTC TTC AAG 3′
IR reverse primer	5′ GTC ACA TTC CCA ACA TCG CC 3′
IR/A probe	5′ TGG GGT TCG AAA AAC C 3′
IR/B probe	5′ GGC ACC AGT GCC TGA AGA GG 3′

**Figure 2:**
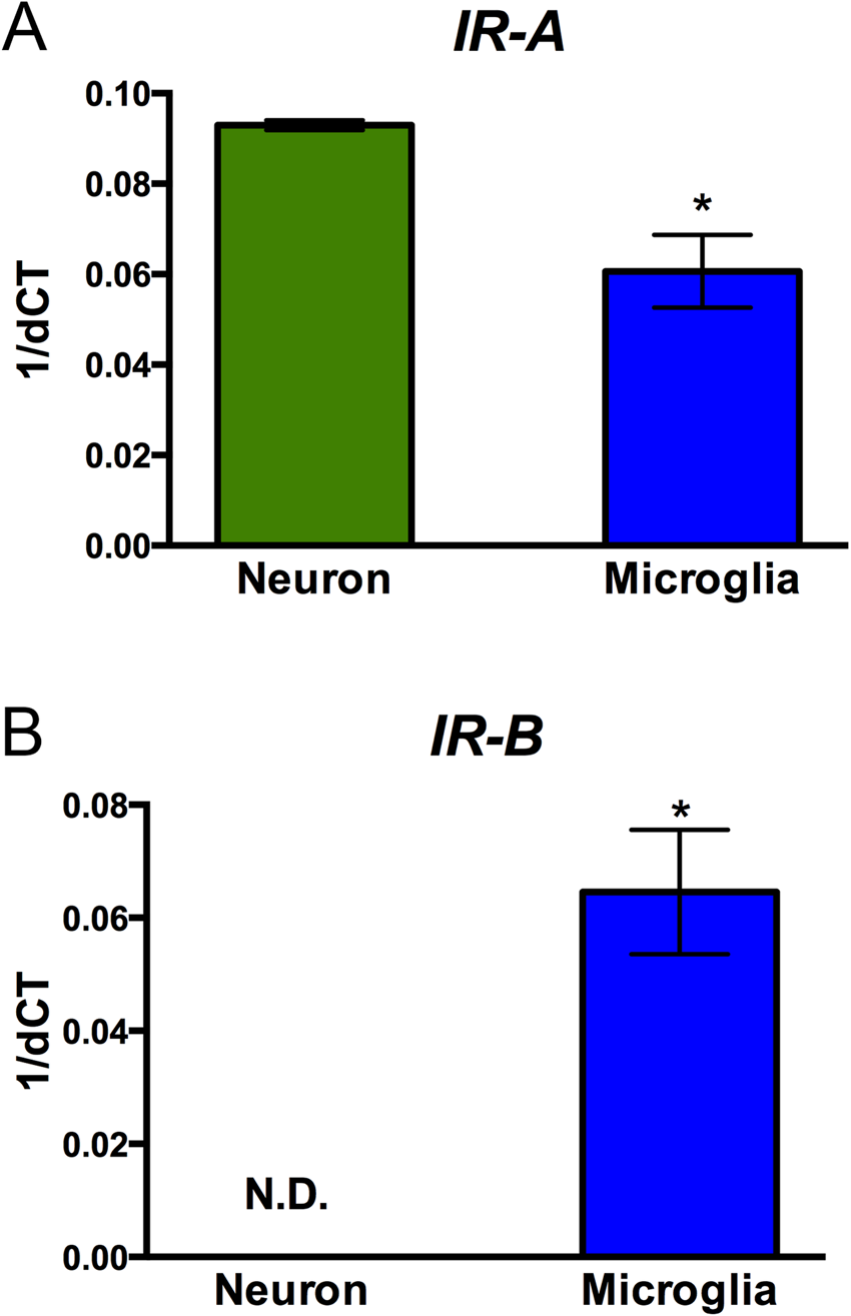
Detection of IR/A and IR/B isoforms in cultures of human neurons and microglia cells by qPCR. Total RNA from differentiated SH-SY5Y neurons and human primary human microglia-SV40 cells were extracted and assayed by qPCR with the insulin receptor primers described in Table 1 and human ß actin internal control primers. Results are expressed as 1/ΔΔCt for (**A**) IR/A and (**B**) IR/B. (**C**) The ratio of IR/A:IR/B was determined from the results of the qPCR. *p < 0.05 compared to neurons.

We first analyzed the expression of insulin receptor isoforms in two brain-derived cell lines using the isoform specific primers for quantitative PCR (qPCR). Human neuronal SH-SY5Y cells differentiated for 7 days with retinoic acid showed expression of IR/A isoform; whereas expression of IR/B was below the level of detection of our system, similar to a previous report of cultured human neuronal cells *in vitro*^9^ (Fig. 2). In contrast, an immortalized primary human microglia-SV40 cell line (ABM), expressed both the IR/A and IR/B isoforms at similar levels as detected by qPCR (Fig. 2).

To test the RT-PCR/ FISH assay on the cultured cells, we next grew them on glass coverslips coated with either collagen (SH-SY5Y) or extracellular matrix (ECM) (human microglia-SV40). Cells were fixed with 4% PFA and then washed with PBS prior to the assay. Cells were first permeabilized with proteinase K for 15 minutes at 37 °C to allow the reverse transcriptase and DNA polymerase enzymes access to the mRNA inside the cells. Next, the slides containing the cells were subjected to RT-PCR with an *in situ* PCR machine with the primers described in Table 1. IR/A and IR/B specific probes were labeled with Alexa 488 and Alexa 594 respectively and hybridized with the cells on the slides in hybridization solution at 48 °C overnight before analysis with a fluorescent microscope.

**Figure 3:**
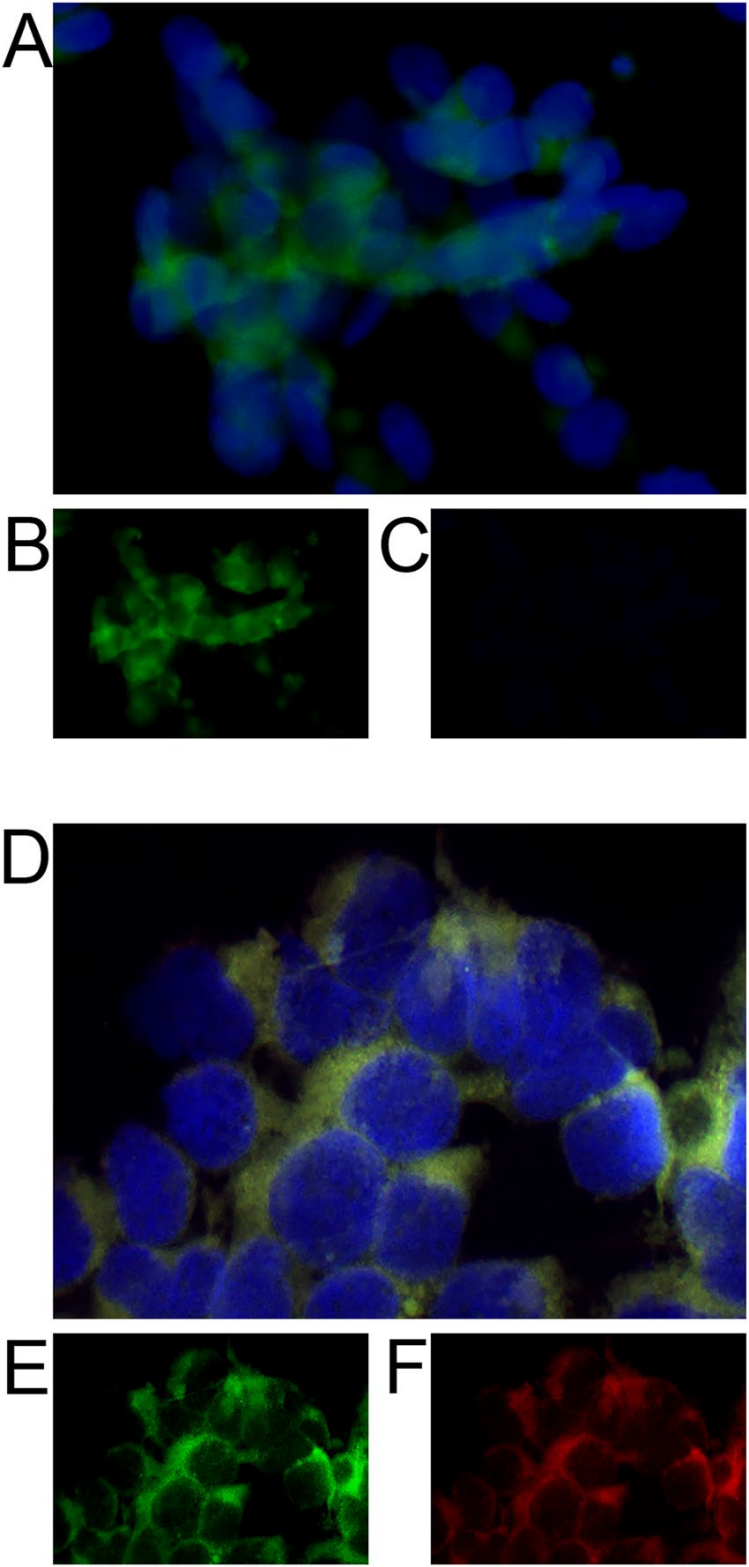
Detection of IR/A and IR/B isoforms *in vitro* in human neurons and microglia cells by *in situ* RT-PCR/FISH. (**A**) SH-SY5Y neuronal cells were differentiated for 7 days on glass coverslips and assayed for (**B**) IR/A (green) and (**C**) IR/B (red). Coverslips were mounted with Vectashield containing DAPI to visualize the nuclei. Scale bar = 20 µm. (**D**) SV40 human microglia cells were grown on glass coverslips and assayed for (**E**) IR/A (green) and (**F**) IR/B (red) and then mounted with Vectashield containing DAPI (blue) to visualize the nuclei. Scale bar = 10 µm.

Compared to control cells that did not undergo RT-PCR or did not receive labeled IR/A or IR/B probe (data not shown), SH-SY5Y neuronal cells showed robust peri-nuclear signal of IR/A but not IR/B as would be expected from the qPCR results (Fig. 3A–C). To determine if we could detect both the IR/A and IR/B transcripts in the same cell, we used the human microglia-SV40 cell line that had shown expression of both isoforms by qPCR. *In situ*/ FISH for IR/A and IR/B detected both transcripts in all cells, with IR/A showing positive green signal and IR/B labeled in red (Fig. 3D–F). Fluorescence was localized to the peri-nuclear region as would be expected for mRNA, similar to that observed for SH-SY5Y cells.

Although real-time qPCR is sufficient to detect IR/A and IR/B transcripts *in vitro* in mono-cellular cultures, detection and localization of the IR/A and IR/B insulin receptor isoforms in tissue is not possible by this method; therefore, *in situ*/FISH was developed primarily for this use. We obtained formalin fixed paraffin embedded (FFPE) human frontal cortex brain tissue from the UCSD Alzheimer’s Disease Resource Center sectioned at 7 µm for analysis by of IR/A and IR/B expression. Following the RT-PCR/FISH protocol, sections were stained by immunohistochemistry with an antibody for MAP2 followed by HRP conjugated secondary and DAB visualization. Slides were mounted with DAPI to visualize the nuclei and were imaged with a digital fluorescent microscope and then analyzed with the co-localization analysis software package plug-in Squassh for Fiji (ImageJ).

**Figure 4:**
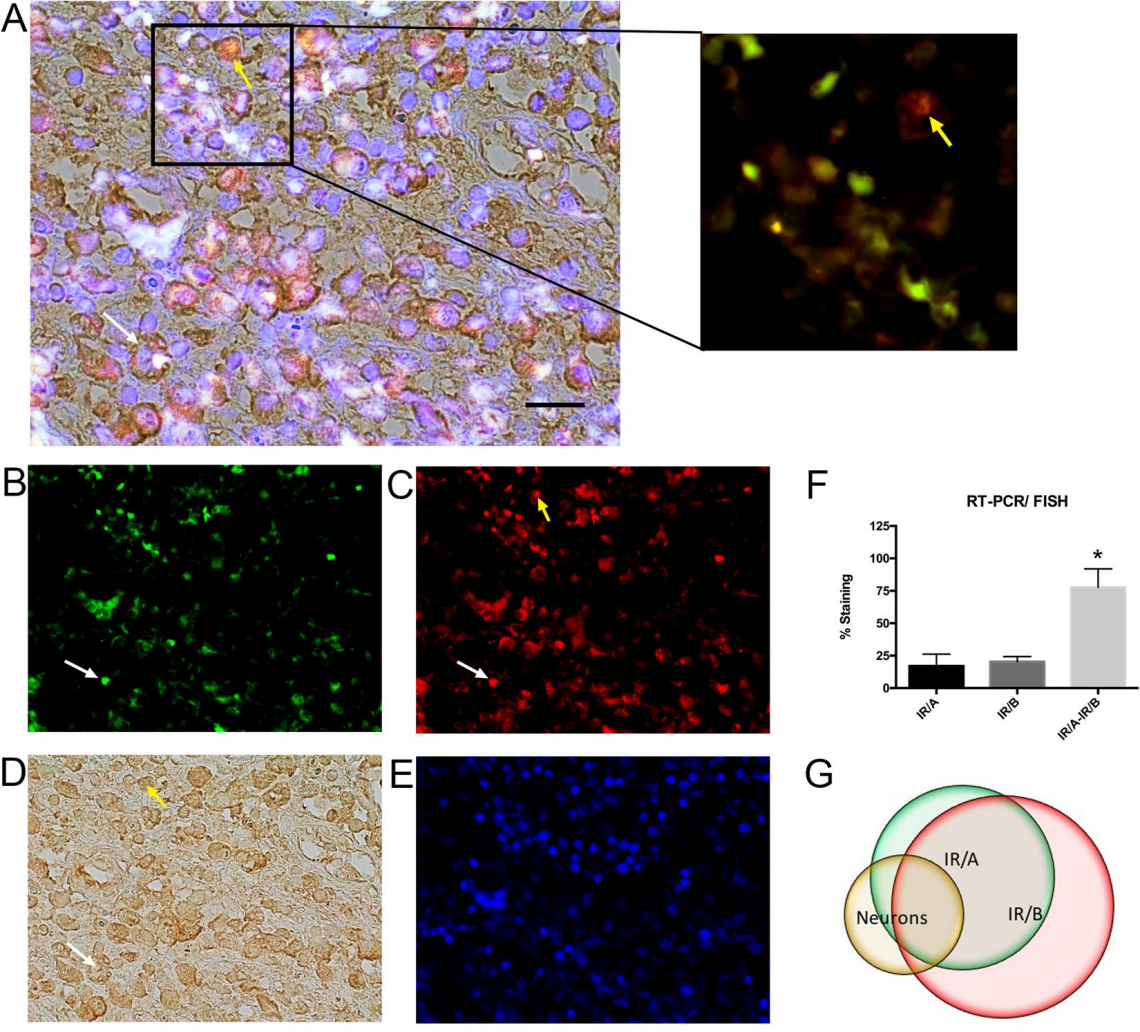
Representative *in situ* RT-PCR/ FISH for IR/A and IR/B along with MAP2 immunohistochemistry in the frontal cortex of the human brain. (**A**) FFPE frontal cortex human brain tissue (7 µm) was assayed by RT-PCR/FISH for (**B**) IR/A (green) and (**C**) IR/B (red) followed by immunohistochemistry for the neuronal marker (**D**) MAP2. Sections were mounted with Vectashield containing (**E**) DAPI (blue) to visualize the nuclei. Breakout of (**A**) shows the co-localization of the red (IR/A) and green (IR/B) signals and specifically a neuron expressing only the IR/A isoform (yellow arrow). (**F**) Staining analysis of IR/A (red) and IR/B (green) signal by digital fluorescent microscopy and Squassh. (**G**) Co-localization analysis of IR/A (green) and IR/B (red) signal with MAP2 (DAB) immunohistochemistry. White arrow indicates co-localization of IR/A, IR/B and MAP2 staining. Yellow arrow indicates co-localization of IR/B only with MAP2 staining. *Indicates statistical significance compared to IR/A or IR/B alone (p < 0.01) using ANOVA with Tukey’s multiple comparison. Scale bar = 25 µm.

Analysis of staining for IR/A and IR/B in the human frontal cortex by RT-PCR/ FISH showed approximately 17% of total cells in the frontal cortex expressed only IR/A, with a similar number of cells expressing only the isoform IR/B (20%) (Fig. 4A–C,F). In contrast, IR/A and IR/B co-localized to 77% of the same cells indicating that co-expression was much more common (Fig. 4A,F). This study also confirmed the specificity of the IR/A by the appearance of individual cells staining for the IR/A isoform alone and not co-labeling for the IR/B isoform (Fig. 4A,F).

Previous studies had examined the expression of IR/A and IR/B in neurons and determined IR/A expression was dominant {Garwood, 2015 #1223}; however, to determine whether neurons in the adult human brain expressed IR/A and/or IR/B, we co-localized the IR/A and IR/B signals to the neuronal MAP2 immunohistochemical staining. A simple mask was applied to the MAP2 stained image and subsequently processed with Image J using the Squassh plug-in to allow the co-localization. Approximately 17% of the MAP2 positive neuronal cells expressed only the IR/A receptor isoform while 50% of neuronal cells expressed both IR/A and IR/B isoforms together (Fig. 4A,G). In contrast, 5% of neurons expressed only the IR/B isoform (Fig. 4A,G). Interestingly, approximately, 25% of cells stained positive for MAP2 but did not show any positive signal for IR/A nor IR/B isoforms. Co-localization of the IR/A and IR/B signals to the same cell was confirmed by laser scanning confocal microscopy (Supplemental Fig. 1). Therefore, neurons in the adult human frontal cortex do express both isoforms of the insulin receptor to varying degrees.

## Discussion

Immunohistochemical analysis of insulin receptor isoforms is difficult to achieve because the difference in size between the two receptors is only 12 amino acids. Analysis by qPCR can be accomplished through the use of isoform specific primers; however, results from this assay cannot be attributed to individual cells in a tissue. We describe a new *in situ* RT-PCR/FISH assay that allows the analysis of both IR/A and IR/B isoforms of insulin receptor as well as localization to cell specific markers in human brain tissue. Thus, for the first time, we present a method that will allow investigators to determine the specific cellular expression levels of IR/A and IR/B isoforms in different human tissues and in different disease states.

Detection of gene expression *in situ* by RNA expression using FISH technology was first reported in the 80’s as a method to track expression of genes in cells without extracting whole RNA from soluble tissue, thus destroying tissue architecture^14^. Recently, advances in FISH technology have allowed increased specificity, decreased false positives and improved accuracy of RNA localization^15^. With the addition of more fluorescent colors and microscope filters, additional RNA molecules can simultaneously be imaged in the same cell. Many of these newer technologies rely on one long probe (40–50nt)^16^ or multiple (30–40) small probes^17^ to visualize a single gene. In the case of insulin receptor isoforms however, the 12 amino acid difference between IR/A and IR/B prevents the use of these technologies since long probes or multiple probes over a long sequence would hybridize to both IR/A and IR/B. Thus, our approach involved the amplification of the two specific messages followed by small, unique labeled probes that bind to IR/A and IR/B differentially.

In this study, we showed IR/A and IR/B expression in the human brain. Insulin receptor isoforms A and B have both been shown to be expressed on human astrocytes^9,18^ and microglia^18,19^ confirming our qPCR results and the results of the *in situ* PCR/FISH assay presented here. Similarly, Garwood *et al*. showed that immortalized human neurons expressed only IR/A *in vitro* as detected by qPCR, in agreement with our results using the human immortalized neuronal SH-SY5Y cell line^9^. In contrast, we observed IR/A and IR/B expression in the MAP2 positive neurons in the human brain by *in situ* RT-PCR/ FISH. This is the first report of IR/B expression in mature neurons. This observation would not have been possible by qPCR on whole brain homogenates as the IR/B signal from surrounding astrocytes and microglia cells would have masked the signal from neurons. The specific localization of the IR/A and IR/B signal to the neurons in the brain section was only possible through RT-PCR/FISH as described in this report.

Previous results have shown insulin stimulation of primary rat hippocampal neurons increases synaptic localization of the insulin receptor via the PI3K/AKT signaling pathway^20^ suggesting signaling through the IR/B isoform and intracellular pathway^21^. In a separated experiment, insulin stimulation of hippocampal neurons activated ATP sensitive K^+^ channels in order to dampen Ca2^+^ oscillations^22^. This last pathway occurred through the MAPK pathway indicating a separate and distinct signaling pathway from that reported in the synaptic upregulation. In fact, the MAPK intracellular signaling pathway suggests signaling occurred through the IR/A isoform^23^. Thus IR/A and IR/B may both be expressed and have distinct functions in neurons.

A neuronal-expressed IR/B isoform has previously been described in invertebrates as an insulin receptor isoform B analogue discovered in C. elegans containing an additional exon between exons 11 and 12, designated DAF-2c, has been shown to be expressed on neurons in the CNS^24^. The DAF-2c receptor is localized to synapses of chemosensory neurons and is involved in learning and memory^24^.

Alterations in the ratio of IR/A to IR/B isoforms have been associated with disease. For instance, increased IR/A:IR/B ratios in muscle are linked to myotonic dystrophy type I (MD1) where the splicing factor CUG-BP1 is abnormally expressed resulting in increased splicing of the IR transcript and higher levels of IR/A mRNA^25–27^. Similarly, increased IR/A:IR/B ratios reported in muscle in myotrophic dystrophy type II (MD2) are associated with increased expression of a CUG repeat-containing RNA that appears to direct splicing^28,29^.

IR/A:IR/B ratios can change with respect to aging too. In rats, increased IR/A:IR/B ratio was linked with increased age^30^ and associated with insulin resistance and glucose intolerance in the liver, adipose tissue and skeletal muscle^31,32^. In contrast, in aged humans (i.e. centenarians) insulin sensitivity, which is also a key feature in long-lived mice^33^, is associated with longevity^34^. Similarly, alterations in insulin receptor signaling have been associated with Alzheimer’s disease^35^ and Parkinson’s disease^36^. Thus, the ratios of IR/A:IR/B in different tissues may lead to clues to age related pathologies or even longevity.

## Methods

### Real-time PCR analysis

Total RNA was isolated from cells using the RNeasy mini kit (Qiagen) and was reverse transcribed using RT^2^ First Strand kit (Qiagen) from 1 μg of total RNA. Quantitative PCR (qPCR) analysis was performed using the StepOnePlus real-time PCR system (Applied Biosystems) with primers described in Table 1. Relative quantification of gene expression was calculated by the comparative threshold cycle (Ct) method and expressed as 2-exp (ΔΔCt) using human ß-actin as an internal control as previously described^37^.

### Preparation of cells

The maintenance and differentiation of human SH-SY5Y neuronal cells were previously described^38^. Adult immortalized human microglia cells-SV40 (Applied Biologics Materials, Inc. Richmond, Canada) were cultured according to manufacturer’s directions. Briefly, cells were maintained in PriGrowIII Media (ABM) containing Pen/Strep (Fisher Scientific) and 10% FBS (Gemini) in 5% CO^2^ and 37 °C. For RT-PCR/ FISH, cells were plated on acid etched glass coverslips coated with extra-cellular matrix (ECM, 1:2 in PBS, Applied Biologics Material #G422) at a density of 1 × 10^5^ cells for 3 days before fixing with 4% paraformaldehyde.

### Preparation of tissue

FFPE human frontal cortex brain tissue from the Shiley Marcos Alzheimer’s Disease Brain Bank at UCSD was sectioned at 7 µm thickness and mounted onto Superfrost slides (Fisher Scientific). Slides were then deparaffinized. Briefly, slides were dipped: twice each in 100% Xylene - 5′, 50%/50% Xylene/ Ethanol - 3′, 100% Ethanol - 3′, 95% Ethanol - 3′, 70% Ethanol - 3′, 50% Ethanol - 3′ and diH_2_O - 3′. Slides were air dried for 1 hour and then stored at −80 °C until used for staining.

### *In situ* RT-PCR/FISH

Detection of the insulin receptor isoforms was carried out similarly to previously published protocols for *in situ* PCR/FISH^39^. PFA fixed cells on glass coverslips were mounted to Superfrost slides (Fisher Scientific) and then a Frame-Seal Incubation Chamber (17 × 28 mm) (Bio-Rad) was added to the slide for incubation procedures. Deparaffinized FFPE tissues already mounted to slides also received a Frame-Seal Incubation Chamber (17 × 28 mm). Cells or tissues were treated with Proteinase K [2 µg/ml in PBS] (Life Technologies) at 37 °C for 15 minutes in an *in situ* PCR machine to permeabilize membranes for the reverse transcriptase and DNA polymerase enzymes. Following the 37 °C incubation, slides were heated to 92 °C for 2′ to inactivate the Proteinase K. Slides were washed once with PBS-T (PBS + 0.05% Tween-20) – 5′ and then once with diH_2_O – 5′.

Following proteinase K treatment, insulin receptor mRNA was amplified by RT-PCR with primers specific for each isoform (Table 1)^13^ using the OneStep RT-PCR Kit (Qiagen). The following parameters were used in the *in situ* PCR machine; 50 °C–45′, 95 °C–15′ for reverse transcription; 30 cycles each of 94 °C–1′, 50 °C–1′, 72 °C–1’ for amplification; 72 °C–10′ for elongation. Following RT-PCR, slides were washed twice with 2 × SSC (30 mM sodium citrate, 300 mM NaCl, pH 7.0) – 5′ and then incubated on a heat block 92 °C–1′.

Probes designed for each isoform (Table 1) were labeled with the ULYSIS Nucleic Acid Labeling Kit 488/594 (Thermo Fisher) and purified with Princeton Separation column (CentriSpin-10, Thermo Fisher) according to manufacturer’s directions. IR/A (125ng) and IR/B (125ng) probes were hybridized in *in situ* hybridization buffer (Enzo) in a plastic dish containing wetted paper towels to generate a humidified environment at 48 °C overnight. After hybridization, slides were washed three times with PBS-T for 5 minutes each and then mounted with anti-fading media containing DAPI (Vectashield, Vector Laboratories) for 3-color imaging with a digital fluorescent microscope (Zeiss). Control slides included no RT-enzyme or no probe (not shown).

### Immunohistochemistry

For cell-specific co-localization, following *in situ* RT-PCR/ FISH, tissues were incubated with anti-MAP2 antibody (Neuron, Millipore) and then incubated with biotinylated secondary antibody and reacted with diaminobenzidine^40^. Slides were mounted with anti-fading media containing DAPI (Vectashield, Vector Laboratories) for 3-color imaging followed by light imaging for DAB staining with a digital fluorescent microscope (Zeiss).

Analysis of co-localization was performed on 5 separate images per condition by Fiji (ImageJ) using the Squassh plugin as previously described^41,42^.

## Electronic supplementary material

Supplementary Figure 1
