# Supplementary material for: Identification of Insulin Receptor Splice Variant B in Neurons by in situ Detection in Human Brain Samples
Source: Sci Rep. 2018 Mar 6;8:4070. doi: 10.1038/s41598-018-22434-2 (PMC5840297; doi:10.1038/s41598-018-22434-2)

Identification of Insulin Receptor Splice Variant B in Neurons by *in situ* Detection in Human Brain Samples

Brian Spencer[1], Logan Rank[1], Jeff Metcalf[2], Paula Desplats[1,2]

Supplemental Figure 1. IR/A and IR/B co-localize to the same cell in the frontal cortex of the human brain. Representative laser scanning confocal microscopy following *in situ* RT-PCR/ FISH for IR/A and IR/B in the frontal cortex of the human brain showing co-localization of IR/A and IR/B signal to the same cell. Scale bar = 10μm.

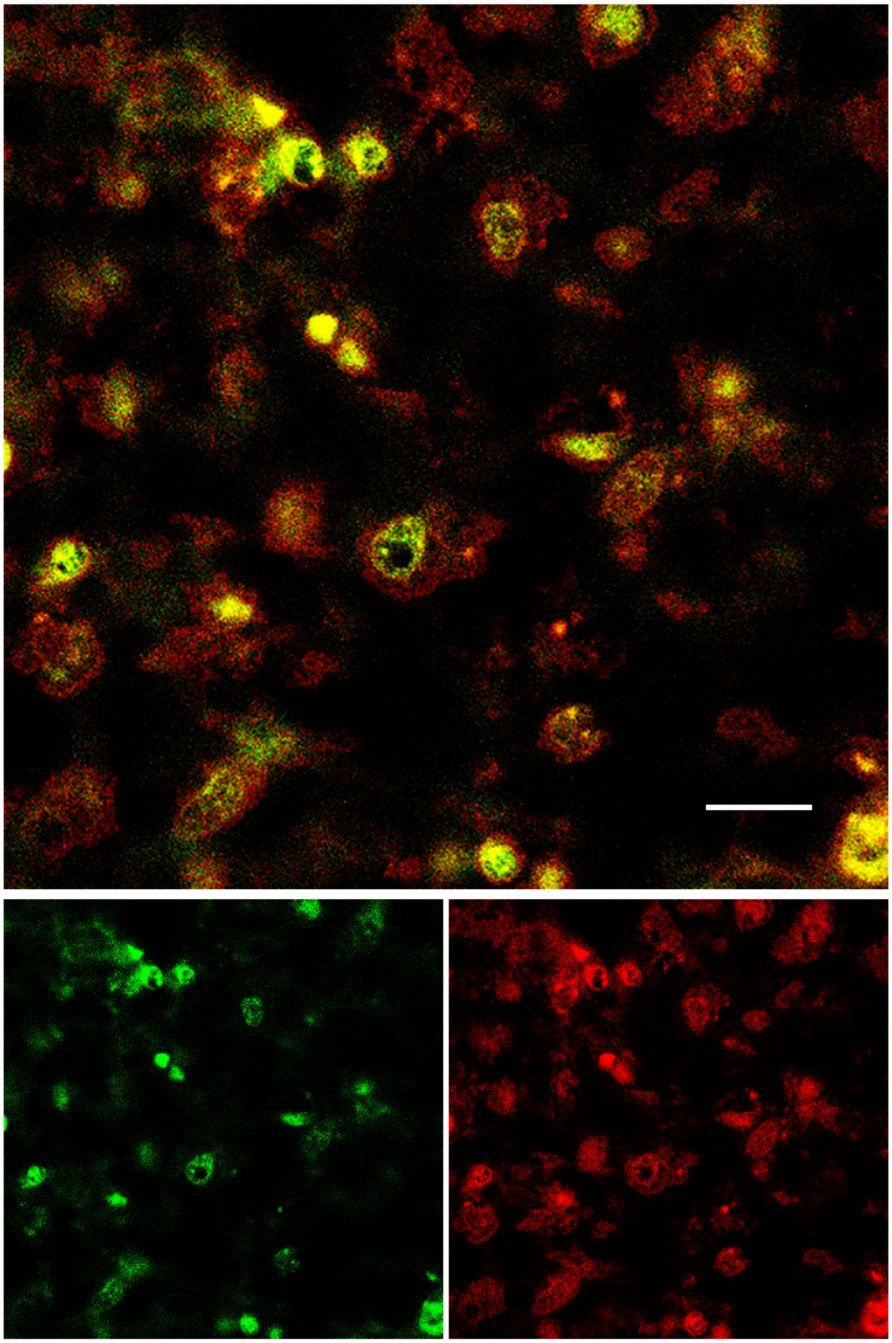

Supplement: Supplementary file 1 — Supplementary Figure 1 [file 41598_2018_22434_MOESM1_ESM.pdf]
